# Unveiling a novel fusion gene enhances CAR T cell therapy for solid tumors

**DOI:** 10.1186/s12943-024-02007-w

**Published:** 2024-05-10

**Authors:** Zefeng Zhou, Yongming Xia, Ruixiu Chen, Panpan Gao, Shiwei Duan

**Affiliations:** 1 Department of hematology, Yuyao People’s Hospital of Zhejiang Province, The Affiliated Yangming Hospital of Ningbo University, Yuyao, Zhejiang 315400 China; 2 Department of Clinical Medicine, Hangzhou City University, Hangzhou, Zhejiang China

**Keywords:** Adoptive cell transfer therapy, CAR T cells, CARD11-PIK3R3, Solid tumors, Cancer immunotherapy

## Abstract

The efficacy of Adoptive Cell Transfer Therapy (ACT) in combating hematological tumors has been well-documented, yet its application to solid tumors faces formidable hurdles, chief among them being the suboptimal therapeutic response and the immunosuppressive milieu within the tumor microenvironment (TME). Recently, Garcia, J. et al. present compelling findings shedding light on potential breakthroughs in this domain. Their investigation reveals the pronounced augmentation of anti-tumor activity in CAR T cells through the introduction of a T cell neoplasm fusion gene, CARD11-PIK3R3. The incorporation of this gene into engineered T cell therapy holds promise as a formidable tool in the arsenal of cancer immunotherapy. The innovative strategy outlined not only mitigates the requirement for high doses of CAR T cells but also enhances tumor control while exhibiting encouraging safety profiles. The exploration of the CARD11-PIK3R3 fusion gene represents an advancement in our approach to bolstering the anti-tumor efficacy of immunotherapeutic interventions. Nonetheless, the imperative for further inquiry to ascertain its transfection efficiency and long-term safety cannot be overstated. Nevertheless, this seminal investigation offers a beacon of hope in surmounting the formidable treatment impediments posed by solid tumors, paving the way for a transformative era in cancer therapeutics.

## Main text

Adoptive Cell Transfer (ACT) therapy stands as a pivotal pillar in cancer treatment, leveraging the innate capabilities of the patient’s immune system to combat malignancies. This therapeutic modality operates by bolstering the patient’s immune response, particularly through the augmentation of T cell function. Broadly, ACT encompasses two primary modalities: T cell receptor (TCR) T cell therapy and chimeric antigen receptor (CAR) T cell therapy. These innovative approaches hold immense promise in addressing select cancer types, notably hematological malignancies, by reprogramming T cells to more adeptly identify and eradicate cancerous cells.

While ACT has demonstrated remarkable efficacy in hematological cancers, its application to solid tumors encounters formidable challenges. Predominant among these hurdles are the limited infiltration capacity of T cells within tumor tissues, diminished persistence and effector function, the immunosuppressive milieu of the tumor microenvironment (TME), treatment-associated toxicity, and antigenic evasion [1]. Consequently, the scientific community is actively engaged in a multifaceted exploration of strategies aimed at amplifying T cell activity, prolonging their survival, and enhancing their tumor-targeting prowess.

These strategies encompass a diverse array of approaches, including immune checkpoint blockade (ICB), activation of costimulatory receptors, transcription factor-based interventions, epigenetic modulation, metabolism-centered interventions, and cytokine-based therapies [2–6]. Each strategy offers distinct mechanisms of action and potential advantages and drawbacks. However, their collective objective is to rejuvenate T cell-mediated anti-tumor responses, thereby offering renewed hope in the battle against cancer.

**Fig. 1:**
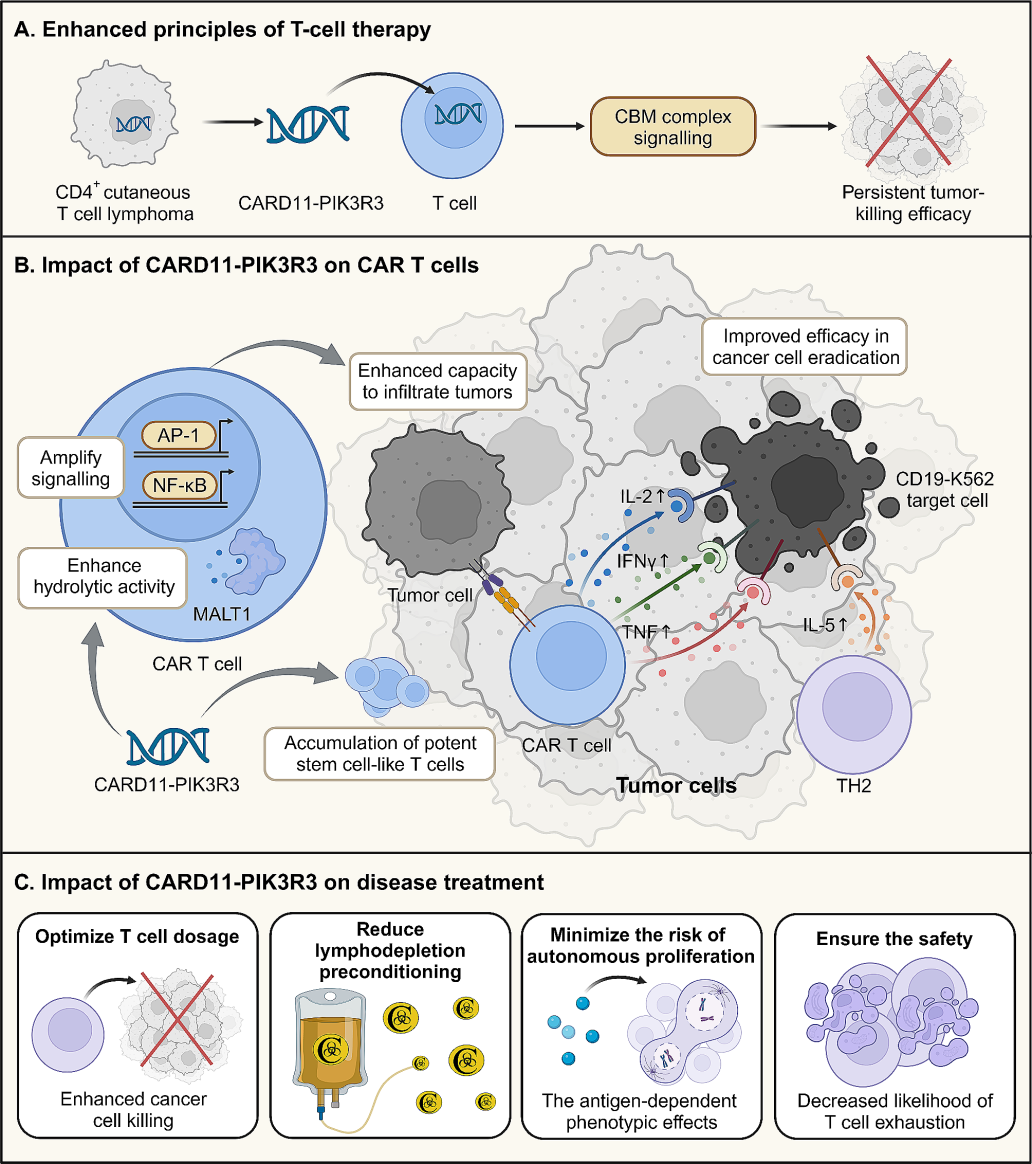
(**A**) Through the incorporation of the naturally occurring CARD11-PIK3R3 fusion gene identified in CD4^+^ cutaneous T cell lymphoma, modified T cell therapy orchestrates a potent enhancement of the CBM complex signaling pathway. This molecular modulation primes T cells, amplifying their tumor-killing prowess. (**B**) CAR T cells endowed with the CARD11-PIK3R3 fusion gene exhibit augmented AP-1 and NF-κB signaling pathways alongside heightened MALT1 proteolytic activity. Consequently, these genetically engineered CAR T cells display elevated secretion of critical cytokines, including IL-2, IFNγ, TNF, and TH2 IL-5, thereby bolstering their proficiency in eradicating cancer cells. (**C**) The therapeutic impact of the CARD11-PIK3R3 fusion gene is manifold, encompassing a reduction in the requisite dosage of T cell therapy, thereby alleviating the necessity for lymphatic clearance pretreatment. Additionally, it mitigates the risk of autonomous proliferation, while importantly maintaining a commendable safety profile

Recent research conducted by two collaborative teams, under the leadership of Julie Garcia and Jay Daniels, has revealed innovative strategies. These strategies harness naturally occurring mutations within tumor cells to enhance CAR T cell therapy. This collaborative effort underscores the combined expertise and leadership of both laboratories in advancing novel approaches to cancer treatment [7]. Their study focused on exploiting the evolutionary adaptations of T cell neoplasms, integrating these unique mutations into CAR T cells to augment their anti-tumor efficacy. Notably, the team identified a gene fusion in CD4^+^ cutaneous T-cell lymphoma, which includes caspase recruitment domain-containing protein 11 (CARD11) and phosphoinositide-3-kinase regulatory subunit 3 (PIK3R3) (Fig. 1A). The researchers meticulously constructed a library comprising 71 mutations closely associated with T-cell tumors, with the explicit aim of delving deeply into the mechanistic underpinnings of each mutation in tumor genesis and progression. Amidst this diverse array of mutations, the CARD11-PIK3R3 fusion gene has garnered considerable attention due to its distinctive biological properties. In vitro experimental findings underscored the fusion gene’s notable capacity to bolster T cell functionality and enhance tumor-killing efficacy significantly. Furthermore, in vivo experiments showcased its remarkable resilience, furnishing robust support for subsequent therapeutic interventions. Consequently, it emerged as a promising candidate for enhancing adoptive cell therapy (ACT) through mutation screening.

The introduction of the CARD11-PIK3R3 fusion gene into CAR T cells demonstrated remarkable enhancements in tumor-targeting capabilities, particularly evident in immunotherapy refractory models. This fusion gene not only facilitated CAR T cell expansion but also elevated cytokine secretion, thereby bolstering cytotoxicity and potentially enhancing long-term therapeutic outcomes, particularly in the context of solid tumors.

Mechanistically, the CARD11-PIK3R3 fusion gene amplified CARD11-BCL10-MALT1 (CBM) signaling in an antigen-dependent manner, thereby potentiating the anti-tumor efficacy of CAR T cells across various immunotherapy-refractory models. This augmentation of signaling pathways, notably NF-κB and AP-1, culminated in heightened interleukin-2 (IL-2) production, essential for sustaining memory phenotypes and bolstering effector functions.

Furthermore, the CARD11-PIK3R3 fusion gene induced significant upregulation of IL-2, interferon-γ (IFNγ), tumor necrosis factor (TNF), and interleukin-5 (IL-5) secretion by CD8^+^ BBz-CAR T cells. Intriguingly, CAR T cells harboring CARD11-PIK3R3 exhibited robust target cell elimination, irrespective of IL-2 levels, underscoring their potent anti-tumor activity (Fig. 1B).

The CARD11-PIK3R3 fusion gene emerges as a transformative component in enhancing the therapeutic efficacy of CAR T cells, facilitating a significant reduction in the requisite dosage of CAR T cells while augmenting their anti-cancer potency. This breakthrough not only diminishes the demand for high CAR T cell doses but also mitigates the necessity for intensive lymphodepletion pretreatment, thereby streamlining therapeutic protocols (Fig. 1C).

In melanoma models, T cells harboring the CARD11-PIK3R3 fusion gene showcased enhanced tumor infiltration capabilities, accompanied by the accumulation of highly functional stem cell-like T cells, culminating in heightened production of TNF, IFNγ, and IL-2. Notably, even at low doses, CARD11-PIK3R3 OT-1 cells exhibited superior tumor growth control. Crucially, the phenotypic effects of CARD11-PIK3R3 are contingent upon antigen presence, minimizing the risk of autonomous proliferation. Furthermore, unlike the potential mutagenic effects associated with lymphocyte-depleting chemotherapy, which may increase the risk of secondary malignant tumors, the application of CARD11-PIK3R3 demonstrates promising potential not only in mitigating T cell exhaustion but also in maintaining a favorable safety profile. During the extended observation period of 418 days, no evident signs of malignant transformation were observed, further validating its safety and efficacy. (Fig. 1C). Nonetheless, on November 28, 2023, the FDA issued a significant notification highlighting the occurrence of T cell malignancies in certain patients undergoing CAR-T cell therapy. In response, the FDA has explicitly mandated that all modified autologous T cell immunotherapies undergo comprehensive long-term safety studies, extending up to 15 years of follow-up, as detailed in their official communication [8]. This rigorous oversight is essential to fully safeguard patient treatment safety and long-term well-being. Given this updated guidance from the FDA, we recognize the imperative for further in-depth evaluation and stringent monitoring of the enhanced CAR-T cell therapy developed by Garcia, J. et al. This proactive approach is crucial not only to ensure the therapy’s safety over extended periods but also to continually refine CAR-T therapy and mitigate potential side effects. By prioritizing ongoing assessment and optimization, we aim to provide patients with safer and more efficacious treatment options. This ongoing commitment to evaluation is paramount to the advancement and enhancement of CAR-T therapy, underscoring our unwavering dedication to protecting patients’ health.

Moreover, recognizing the intricate landscape of the tumor microenvironment, a combinatorial approach integrating the CARD11-PIK3R3 fusion gene with other immunotherapeutic modalities, such as immune checkpoint blockade (ICB) and costimulatory receptor activation, warrants exploration to engender synergistic anti-tumor effects.

In essence, adoptive cell therapy heralds immense promise in cancer therapeutics, particularly underscored by the triumphs witnessed in CAR T cell and TCR T cell therapies for hematological malignancies. Despite the complexities inherent to solid tumor treatment, relentless scientific inquiry and technological innovation, epitomized by the discovery of the CARD11-PIK3R3 fusion gene, furnish renewed strategies and optimism for surmounting these challenges. Moving forward, concerted efforts in research and clinical trials hold the potential to usher in safer and more efficacious treatments, heralding newfound hope for cancer patients worldwide.

## Data Availability

All relevant data are within the paper and its Supporting Information files.
